# Possible Positive Selection for an Arsenic-Protective Haplotype in Humans

**DOI:** 10.1289/ehp.1205504

**Published:** 2012-10-16

**Authors:** Carina M. Schlebusch, Cecil M. Lewis, Marie Vahter, Karin Engström, Raúl Y. Tito, Alexandra J. Obregón-Tito, Doris Huerta, Susan I. Polo, Ángel C. Medina, Tom D. Brutsaert, Gabriela Concha, Mattias Jakobsson, Karin Broberg

**Affiliations:** 1 Department of Evolutionary Biology, Evolutionary Biology Centre, Uppsala University, Uppsala, Sweden; 2 Department of Anthropology, University of Oklahoma, Norman, Oklahoma, USA; 3 Section for Metals and Health, Institute for Environmental Medicine, Karolinska Institutet, Stockholm, Sweden; 4 Divison of Occupational and Environmental Medicine, Lund University, Lund, Sweden; 5 Facultad de Medicina, Centro de Investigación de Bioquímica y Nutrición, Universidad Nacional Mayor de San Marcos, Lima, Peru; 6 Universidad de Altiplano, Puno, Peru; 7 Department of Exercise Science, Syracuse University, Syracuse, New York, USA; 8 National Food Administration, Risk Benefit Assessment Department, Uppsala, Sweden; 9 Science for Life Laboratory, Uppsala University, Sweden

**Keywords:** *AS3MT*, dimethylarsinic acid, DMA, drinking water, Human Genome Diversity Project, methylarsonic acid, MMA

## Abstract

Background: Arsenic in drinking water causes severe health effects. Indigenous people in the South American Andes have likely lived with arsenic-contaminated drinking water for thousands of years. Inhabitants of San Antonio de los Cobres (SAC) in the Argentinean highlands generally carry an *AS3MT* (the major arsenic-metabolizing gene) haplotype associated with reduced health risks due to rapid arsenic excretion and lower urinary fraction of the monomethylated metabolite.

Objectives: We hypothesized an adaptation to high-arsenic living conditions via a possible positive selection for protective *AS3MT* variants and compared *AS3MT* haplotype frequencies among different indigenous groups.

Methods: Indigenous groups we evaluated were *a*) inhabitants of SAC and villages near Salta in northern Argentina (*n* = 346), *b*) three Native American populations from the Human Genome Diversity Project (HGDP; *n* = 25), and *c*) five Peruvian populations (*n* = 97). The last two groups have presumably lower historical exposure to arsenic.

Results: We found a significantly higher frequency of the protective *AS3MT* haplotype in the SAC population (68.7%) compared with the HGDP (14.3%, *p* < 0.001, Fisher exact test) and Peruvian (50.5%, *p* < 0.001) populations. Genome-wide microsatellite (*n* = 671) analysis showed no detectable level of population structure between SAC and Peruvian populations (measure of population differentiation *F*_ST_ = 0.006) and low levels of structure between SAC and HGDP populations (*F*_ST_ < 0.055 for all pairs of populations compared).

Conclusions: Because population stratification seems unlikely to explain the differences in *AS3MT* haplotype frequencies, our data raise the possibility that, during a few thousand years, natural selection for tolerance to the environmental stressor arsenic may have increased the frequency of protective variants of *AS3MT*. Further studies are needed to investigate this hypothesis.

The widespread occurrence of arsenic-resistance genes in bacteria and archaea, and in eukaryotes such as yeast and plants (Rosen 1999; Song et al. 2010), reflects the fact that arsenic is a ubiquitous environmental toxic metal. Possibly arsenic toxicity has been a selection pressure during evolution, but its role in human evolution is not known.

Increased concentrations of inorganic arsenic (above the World Health Organization guideline value of 10 µg/L) in drinking water are frequently found in Argentina, Bangladesh, Chile, China, Hungary, India, Mexico, Romania, Taiwan, and different parts of the United States [International Agency for Research on Cancer (IARC) 2004; Nuckols et al. 2011; Sanders et al. 2012]. Arsenic contamination occurs mainly through leakage from arsenic-containing bedrock and sediment into the drinking water (mainly groundwater). In most areas, human exposure is a relatively recent occurrence in evolutionary terms, but in a few regions of the world, such as the Andes highlands, people have lived with arsenic-contaminated drinking water for thousands of years as a consequence of natural reservoirs and modern and Pre-Columbian mining activities (Núñez et al. 1991). Studies on ancient Andean mummies buried in northern Chile up to 7,000 years ago have revealed high arsenic concentrations in their internal organs and hair (Pringle 2009).

Arsenic exposure via drinking water is associated with a number of adverse health effects, starting in early life with increased morbidity and mortality (Rahman et al. 2010a, 2010b) and continuing throughout life with increased risks of cancer, vascular diseases, hepatotoxicity, and diabetes (Del Razo et al. 2011; IARC 2004; Sohel et al. 2009). However, there seems to be wide variation in susceptibility to arsenic toxicity. One important susceptibility factor is the efficiency of arsenic metabolism and the rate of urinary excretion of arsenic metabolites.

Inorganic arsenic is metabolized in the body by a series of reduction and methylation reactions, first producing methylarsonic acid (MMA), then dimethylarsinic acid (DMA), both of which are excreted in the urine (Vahter 2002). The most toxic metabolite is the trivalent MMA (Piatek et al. 2008), and although there is some concern about the toxicity of trivalent DMA as well (Naranmandura et al. 2011), the fraction of total MMA in urine is used as a marker for susceptibility to arsenic-related toxic effects (Lindberg et al. 2008): the higher urinary MMA fraction, the more toxic effects.

The main methyltransferase in arsenic metabolism is arsenic (+3 oxidation state) methyltransferase (AS3MT), which can methylate both inorganic arsenic and MMA (Lin et al. 2002). In humans, efficient methylation from inorganic arsenic to DMA is associated with a high rate of arsenic excretion in urine (Gardner et al. 2010; Vahter 2002), which means that there are lower tissue concentrations of arsenic with more efficient methylation.

The distribution of arsenic metabolites in human urine is 10–30% inorganic arsenic, 10–20% MMA, and 60–70% DMA, but there is large variation between individual persons, even after accounting for variation in arsenic exposure (Vahter 2002). A unique exception is the low urinary excretion of MMA among indigenous populations in the Andes, including residents of the Argentinean village of San Antonio de los Cobres (SAC) (Hopenhayn-Rich et al. 1996; Vahter et al. 1995). This difference in arsenic metabolism between populations is partly explained by genetic factors: It was recently shown that six noncoding single nucleotide polymorphisms (SNPs) in *AS3MT* that were associated with altered *AS3MT* gene expression had a strong impact on arsenic metabolism in a population living in the Argentinean Andes highlands and in a population in Bangladesh (Engström et al. 2011). This further lends support to the role of *AS3MT* in arsenic methylation. However, the *AS3MT* haplotype associated with efficient methylation (i.e., less MMA and more DMA in urine) was much more frequent among people living in SAC and the surrounding villages in the Andes highlands compared with other populations studied, such as those in Bangladesh and Europe (Schlawicke Engström et al. 2007). This suggests the hypothesis that genetic selection for *AS3MT* haplotypes associated with a more efficient arsenic metabolism has occurred in populations that have lived in areas with elevated arsenic exposure for thousands of years.

In the present study, we compared the frequencies of inferred *AS3MT* haplotypes for three SNPs associated with arsenic metabolism (Engström et al. 2011) between a group of Argentinean persons predominantly living in a region with high arsenic content in their drinking water and other Native American groups. These groups included three Native American populations from the Human Genome Diversity Project (HGDP) panel and groups from different parts of Peru. We also compared the frequencies of inferred *AS3MT* haplotypes in the Argentinean group to eight East Asian populations from the HGDP panel (Cann et al. 2002). Because population structure among Native American groups potentially can explain differences in haplotype frequencies, we also genotyped 671 autosomal microsatellites in these groups to investigate levels of genetic differentiation.

## Materials and Methods

*Population groups.* Argentina. The SAC study site (3,800 m above sea level) is in the Puna region of the Andes highlands. In this area, arsenic in the volcanic bedrock is released into the groundwater that is used as drinking water and there are no anthropogenic arsenic exposure sources, such as mining, that affect the water. The drinking water in SAC contains about 200 µg arsenic/L, with small variations over time (Concha et al. 2006). We also included previously studied (Concha et al. 1998) persons from villages near Salta (170–400 km east of SAC), the main town in this region of northern Argentina. The participants were from Rosario de Lerma (< 1 µg arsenic/L), Joaquin V. Gonzales (6 µg arsenic/L), and Taco Pozo (about 200 µg arsenic/L; Chaco region).

The people in SAC and the surrounding villages are mainly of Atacameño descent. The Atacameños, who once occupied northern Chile and southwestern Argentina, have lived in the region for 11,000 years (Núñez et al. 1991). There are traces of human settlements in northern Argentina, the Puna area where SAC is situated, from 1,500 BCE (Normando Cruz 2011). In total, 323 participants from SAC and 23 from villages near Salta were sampled in 1994, 1996–1997, 2004–2005, and 2008. There was no overlap among participants from the different sampling occasions. Water and urine samples were obtained for determination of arsenic exposure and metabolite pattern, and blood or buccal swabs were collected for DNA extraction (Engström et al. 2011; Schlawicke Engström et al. 2007). The families of the participants had lived in the area for at least two or three generations according to personal interviews. The SAC study subjects were mainly of indigenous (Atacameño) origin with small (but varying) ancestry from Hispanics. In the villages near Salta, there was greater Hispanic influence. First-degree relatives were excluded from the analysis. All the study subjects drank tap water exclusively. Genotyping data for *AS3MT* in women who participated in 1997 and 2004–2005, and in nonpregnant women who participated in 2008, has been published previously (Engström et al. 2011; Schlawicke Engström et al. 2007).

Men in SAC and the other villages of the Argentinean study populations were often away from home for longer periods for work, and thus had a different pattern of exposure to arsenic. They were therefore not included in the analysis of genetic effects on the metabolism. For comparison of *AS3MT* haplotype frequencies between different populations, both men and women were included, because we did not consider it likely that positive selection would operate differently for the arsenic metabolism phenotype between the sexes.

HGDP populations. Samples for assessing worldwide genetic diversity were collected for the HGDP, and the provided cell lines are maintained at the Centre d’Étude du Polymorphisme Humain (Fondation Jean Dausset-CEPH, Paris, France) for use in population genetic studies (Cann et al. 2002). A previous study (Jakobsson et al. 2008) genotyped some 500,000 SNPs in a broad subset of 485 participants from the HGDP–CEPH and included 25 Native American participants (7 Piapaco from Colombia: geographic coordinates 3°N, 68°W; 10 Maya from Mexico: 19°N, 91°W; and 8 Pima from Mexico: 29°N, 108°W) who represent Native Americans in this study. Additionally, six East Asian populations [Cambodian (*n* = 10), Daur (10), Lahu (8), Mongola (9), Yakut (15), and Yi (10)] were also included and analyzed separately. No urine samples were collected from the HGDP populations.

Peru. The Peruvian study subjects (*n* = 97) were students at the University of San Marcos in Lima; participants who identified themselves as second-generation Quechua migrants currently living in Lima; participants currently living in the Andean highland cities of Cerro de Pasco and Huancayo; and participants from a village near Pucallpa in the jungle area of Peru. The Peruvians were mestizos determined to have predominately native ancestry. No urine samples were taken from the Peruvian subjects.

The samples from the different populations were collected with informed consent (oral and written). The protocol was approved by the Ministry of Health (Salta, Argentina) and the ethics committees of the Karolinska Institutet (Stockholm, Sweden), the University of Oklahoma (Norman, OK, USA), and the Universidad Nacional Mayor de San Marcos (Lima, Peru).

*Arsenic analysis.* Exposure to inorganic arsenic was assessed by the concentration of arsenic in water and total arsenic in urine, that is, the sum of inorganic arsenic, MMA, and DMA. Speciation of arsenic metabolites in urine was performed using HPLC hyphened with hydride generation and inductively coupled plasma mass spectrometry (Agilent 1100 series system, Agilent 7500ce; Agilent Technologies, Waldbronn, Germany), employing adequate quality control (Schlawicke Engström et al. 2007). Arsenic concentrations were adjusted to the mean specific gravity as measured by a hand refractometer (Atago, Tokyo, Japan).

*Genotyping.* The women from Argentina were either genotyped for *AS3MT* SNPs by TaqMan SNP Genotyping (Applied Biosystems, Foster City, CA, USA) or by Sequenom™ SNP Genotyping (Sequenom, San Diego, CA, USA) according to the manufacturer’s protocol. The Peruvian participants were genotyped by TaqMan assays for rs3740393 (C/G, ancestral allele denoted first), rs3740390 (C/T), and rs10748835 (A/G), each containing a protective allele (C, T, and A, respectively) associated with less MMA and more DMA (i.e., a more beneficial metabolism) (Engström et al. 2011). These SNPs are in strong linkage disequilibrium with five other SNPs distributed over approximately 30,000 base pairs along the *AS3MT* gene that also have been associated with arsenic metabolism. The non-synonymous rs11191439, which has been associated with less efficient metabolism, is very rare in this population (2%).

*Imputations.* Genotype imputation is a technique that allows accurate estimation of associations with genetic markers that are not directly genotyped. When a particular stretch of a chromosome is examined in at least one participant, the genotypes of many others who inherit that same stretch of markers are also identified. We analyzed SNPs in linkage disequilibrium with other neighboring SNPs in the *AS3MT* region that could reliably be used to impute genotypes of SNPs that were not genotyped. The HGDP panel has previously been genotyped for a panel of genome-wide SNPs (Jakobsson et al. 2008). This panel did not include the three *AS3MT* SNPs rs3740393, rs3740390, and rs10748835. However, three other *AS3MT* SNPs were present in the panel (rs10509760, rs17115203, and rs1046778), and these SNPs were used to impute the three untyped SNPs associated with arsenic metabolism for the three Native American populations and eight East Asian populations typed by Jakobsson et al. (2008). Similarly, we imputed the three SNPs that were typed in Jakobsson et al. (2008) for the SAC and Peruvian participants. This yielded 6-SNP haplotypes for the HGDP Native Americans and East Asians, SAC, and Peruvian participants [see Supplemental Material, Table S1 (http://dx.doi.org/10.1289/ehp.1205504)]. For imputation, we used a reference panel from the HapMap Phase 2 Project (International Haplotype Map Project 2005) in which all six SNPs were typed. The Japanese (JPT) and Han Chinese (CHB) HapMap data sets were selected as a reference panel for imputation and phasing. For imputation of the unknown variants in Native Americans, we used East Asians as a reference panel, which as such has been shown to perform very well in previous studies (Huang et al. 2009, 2011). Imputation and phasing were performed simultaneously with the PHASE version 2.1 software package (Stephens et al. 2001) but in two separate groups for the HGDP and SAC/Peru populations because the groups were missing different sets of three SNPs. The HapMap reference panel haplotypes were marked as “known phase” in each case and the option to output population-based haplotype frequencies was applied in PHASE. To compare arsenic-protective haplotypes between various groups in the study, 6-SNP haplotypes (see Supplemental Material, Table S1) were reduced to 3-SNP haplotypes (see Supplemental Material, Table S2) by combining frequencies of haplotypes that were identical when only the three protective SNPs were considered.

*Microsatellite analysis.* Fifteen participants from the SAC group and 15 from each of the five sampled Peruvian groups (total *n* = 90) were selected to be typed by PreventionGenetics (http://www.preventiongenetics.com) for 806 short tandem repeat polymorphic markers. Participants included in the microsatellite analysis had sufficient DNA of good quality (260:280 ratio > 1.8) for analysis, were born in the study area, and were not first-degree relatives of other participants included in the analysis (based on self-report). The marker data were integrated with previously published data following the procedure described by Wang et al. (2007), resulting in 671 overlapping microsatellites.

Relationships between pairs of participants were inferred with Relpair version 2.0.1 (Epstein et al. 2000) and first- and second-degree relatives were excluded from further analyses, including one person from SAC and two from Peru. Using the 671 microsatellites, we inferred population structure for the Peruvian (*n* = 73), SAC (*n* = 14), and an expanded set of participants from the three HGDP Native American populations (Wang et al. 2007) (*n* = 42: 7 Piapaco, 14 Pima, 21 Maya).

To estimate population differentiation, we calculated pairwise *F*_ST_ estimates (Wright’s measure of population subdivision; Weir and Cockerham 1984) between each population pair using Genepop version 4.0 (Rousset 2008). We also inferred population structure for the study populations using the clustering software STRUCTURE (Falush et al. 2003). To determine the level of African and European admixture among the Native American participants, we used a supervised clustering approach in which African and European populations from the HGDP sample set were fixed as reference populations. We used the admixture model with the F model of correlated allele frequencies across clusters for the STRUCTURE analysis. Each replicate STRUCTURE run had a burn-in period of 20,000 iterations followed by 20,000 iterations from which estimates were obtained. We repeated the STRUCTURE analysis 10 times for each choice of number of clusters (*K*), from *K* = 3 to *K* = 10. The 10 replicates for each choice of K were summarized using CLUMPP (Jakobsson and Rosenberg 2007) with the Large K Greedy algorithm (10,000 random permutations) to identify common modes among replicates and to combine the clustering results across replicates. The combined clustering result was visualized with DISTRUCT (Rosenberg 2004).

## Results

*Comparison of* AS3MT *haplotype frequencies.* Most of the Argentinean study subjects were women (96% of the participants from SAC; 100% from near Salta), with a median age of 31 years (range 14–76 years; SAC) and 32 years of age (18–53 years; Salta). Fourteen percent of the women in SAC were pregnant. Median (range) arsenic concentrations in urine were 268 µg/L (37–1,250) for participants from SAC and 19 µg/L (3.0–606) for those from near Salta.

The most frequent *AS3MT* haplotype in SAC was C-T-A (rs3740393, rs3740390, rs10748835), which was found in 68.7% of the evaluated participants (Table 1). The C-T-A haplotype was significantly less common in the Native American populations from HGDP (14.3%, Fisher exact test *p* = 2.20 × 10^–16^), the Peruvians (50.5%, *p* = 5.18 × 10^–6^), and the 23 participants who lived near Salta (36.7%, *p* = 2.95 × 10^–5^). This most frequent *AS3MT* haplotype appeared to have a strong effect on the arsenic metabolite pattern in urine in the SAC group [see Supplemental Material, Table S3 (http://dx.doi.org/10.1289/ehp.1205504)]: Increasing copies of the C-T-A haplotype were associated with a smaller percentage of MMA (9.9, 8.8, and 7.0% MMA with 0, 1, or 2 copies, respectively; *p* < 0.001) and a higher percentage of DMA (75.4, 77.2, and 81.0%; *p* < 0.001).

**Table 1:** Inferred *AS3MT* haplotype frequencies (%) in SAC, HGDP Native American, and Peruvian populations.

Haplotype	SAC	Near Salta	HGDP–Native Americans	Peru
n	323	23	25	97
C-T-A	68.7	36.7	14.3	50.5
G-C-G	25.8	36.4	66.8	39.7
G-C-A	4.1	17.9	18.8	7.7
C-T-G	0.6	0.3	0.0	2.1
C-C-A	0.4	3.9	0.0	0.0
G-T-A	0.2	4.0	0.0	0.0
C-C-G	0.2	0.5	0.0	0.0
G-T-G	0.0	0.4	0.0	0.0
Protective alleles are underlined.								

The C-T-A haplotype in SAC was also higher than the inferred frequencies in the East Asian (range = 8.1–37%), Native American HGDP (12–17%), and the European populations [5.8% in the HapMap CEU (Utah residents with ancestry from northern and western Europe) group] [see Supplemental Material, Table S2 (http://dx.doi.org/10.1289/ehp.1205504)]. The phased and imputed haplotypes (both 3-SNP and 6-SNP haplotypes) in HGDP East Asians compared well to the known haplotypes in the HapMap JPT and CHB groups (Supplemental Material, Tables S1 and S2), and it was assumed that phasing and imputation were likely to be reasonably accurate for Native Americans too.

The G-C-G haplotype, which contains no protective alleles, was the second most frequent in the SAC population (26%) (Table 1), but it was the most frequent HGDP Native American haplotype (67%; *p* = 5.16 × 10^–10^ compared with SAC), and occurred at intermediate frequency in Peruvians (40%; *p* = 0.000294 compared with SAC) and in the population near Salta (36%; *p* = 0.119 compared with SAC).

*Population structure analysis of Native American groups.* Pairwise *F*_ST_ based on the 671 microsatellites showed low levels of differentiation between SAC and Peruvian populations (*F*_ST_ = 0.006) and slightly higher levels of differentiation between SAC and the three Native American HGDP groups (*F*_ST_ 0.012–0.054) (Table 2).

**Table 2:** Pairwise comparison of FST between different Native American groups and European and African populations.^*a*^

SAC	Peruvian	Piapoco	Maya	Pima	European	African
SAC	—
Peruvian	0.006	—
Piapoco	0.032	0.027	—
Maya	0.012	0.008	0.029	—
Pima	0.054	0.047	0.077	0.046	—
European	0.062	0.058	0.079	0.056	0.090	—
African	0.080	0.078	0.094	0.073	0.103	0.043	—
aPairwise population FST estimates were calculated according to Wright’s measure of population subdivision (Weir and Cockerham 1984) and showed low levels of differentiation between SAC and Peruvian populations and slightly higher levels between SAC and the three Native American HGDP groups.														

**Figure 1:**
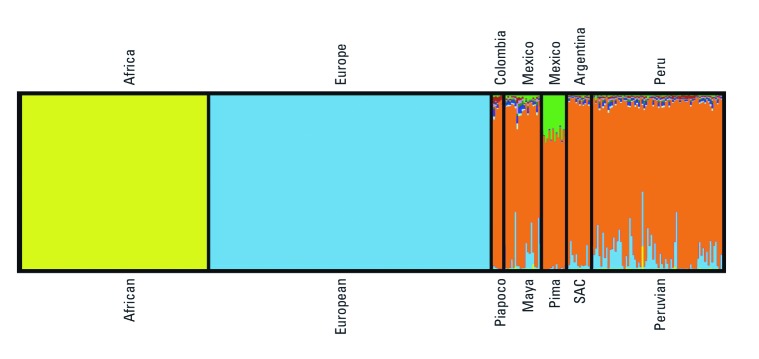
Supervised clustering of groups from SAC, Peru, and HGDP groups. Clustering at *K* = 10 clusters are shown. African and European participants from the HGDP panel were predefined as reference groups for the “yellow” and the “blue” clusters, respectively, in order to determine the European and African admixture levels among the Native American populations. The remaining variation belongs to the main Native American cluster (orange). The Pima showed partial contribution from another group (green) compared with the SAC and the Peruvian populations for *K* = 7 to *K* = 10. Each individual is represented by a vertical line divided into K colors, with each color representing a cluster. Each different population is separated by a black line and labeled by population and location.

The results from a supervised STRUCTURE analysis (Figure 1) demonstrated no detectable level of population structure between the SAC group and the Peruvians. Each participant was probabilistically assigned to a certain number of allowed clusters (*K*), whereas the variation attributable to European and African contribution were fixed to clusters 1 and 2 and the remaining clusters were available to represent remaining population structure in the data set through hierarchical clustering. Although the SAC and Peruvian populations showed slight differences in contribution from the European cluster (blue) and the African cluster (yellow), the remaining variation belongs to the main Native American cluster (orange). This cluster is constant from *K* = 3 to *K* = 10, with no additional clustering within the main Native American cluster. Among the HGDP Native American populations, the Maya and the Piapoco fell in the same cluster (orange) as SAC and the Peruvian populations. The Pima showed partial contribution from another group (green) compared with the SAC and the Peruvian populations for *K* = 7 to *K* = 10.

## Discussion

The findings from this study raise the possibility that haplotypes in *AS3MT*, associated with a more efficient arsenic metabolism and a probably lower formation of the most toxic metabolite, may have undergone positive selection in populations who have lived for a very long time in areas with high arsenic concentrations in the drinking water. This study presents a number of findings that are in support of the hypothesis for positive selection:

We observed significant differences in the frequencies of *AS3MT* haplotypes between SAC participants versus Peruvians and Native Americans from the HGDP panel.

The haplotype that contained three protective alleles (C-T-A), previously associated with more efficient arsenic methylation (Engström et al. 2011), was significantly more frequent in SAC compared with Peruvian groups and HGDP Native Americans, whereas the haplotype containing no protective alleles (G-C-G) was significantly lower in frequency in SAC.

The difference in *AS3MT* haplotypes between SAC and villages near Salta may reflect the more pronounced Hispanic genetic influence in the latter groups and, thus, these populations have been exposed to arsenic only during the last few centuries.

The absence of population structure between the Peruvian groups and SAC (and the low levels of population structure between SAC and the HGDP Native American groups) indicates that the difference cannot be explained by population differentiation attributed to genetic drift.

The differentiation (measured as *F*_ST_) between SAC and Peruvian groups was about 10 times as large for the *AS3MT* gene (0.053) compared with the genome-wide average (0.006; note that these *F*_ST_ values were computed for SNPs and microsatellites and may not be directly comparable).

Possibly, the frequency differences in arsenic-protective haplotypes between these groups may be due to selection for haplotypes containing the protective alleles. Although further studies are needed to confirm this hypothesis, this is, to our knowledge, the first study suggesting human adaptation to a toxic compound.

The mechanism for genetic selection may be via adverse effects of arsenic that occur before reproductive age. Studies of mice and children have showed that arsenic affects the immune system (Ahmed et al. 2010; Fry et al. 2007; Kozul et al. 2009), and increases infant morbidity and mortality (Rahman et al. 2010a, 2010b), which probably reduces fitness, that is, both the ability to survive and to reproduce. Arsenic exposure during pregnancy has been shown to enhance placental inflammatory responses, reduce placental T cells, and to alter cord blood cytokine expression levels (Ahmed et al. 2010). In Bangladesh, the risk of lower respiratory tract infections and diarrhea in infants (Rahman et al. 2011) was shown to increase 69% and 20%, respectively, in children exposed to high, compared with children exposed to low, arsenic concentrations. The rate of infant mortality also increased with increasing arsenic exposure: the hazard ratio was 5.0 (95% CI: 1.4, 18) in the fifth quintile of arsenic exposure (> 268 µg/L), compared with the first quintile (< 33 µg/L) (Rahman et al. 2010a). Children with a slower metabolism, and thus with more toxic metabolites formed, may be more susceptible to arsenic toxicity. However, the effects of the efficiency of the arsenic metabolism in the children were not assessed in the above-mentioned studies. Selection for a protective *AS3MT* haplotype could also be caused by detrimental effects of arsenic occurring somewhat later in life, such as hepatoxicity, cardiovascular disease and impaired lung function (Smith et al. 2006) that result in reduced reproduction. Considering the severe adverse health effects of arsenic both in children and adults, persons who had the arsenic-tolerance haplotype could have had a very strong selective advantage in arsenic-rich environments. An interesting observation from SAC and villages in the area with arsenic-contaminated water is that the commonly occurring skin effects of arsenic (i.e., hyperkeratosis and pigmentation changes) are not prevalent; in fact, we did not observe a single case among approximately 400 examined participants [(Engström et al. 2011; Schlawicke Engström et al. 2007) and authors’ unpublished data]. We believe that the high frequencies of the *AS3MT* haplotype mainly reflect historical selection, because although arsenic exposure is still present in SAC (Engström et al. 2011), there have been improvements in living conditions and health care for children and adults in this area during modern times. However, ongoing selection cannot be ruled out. There is evidence that *AS3MT* SNPs in the protective haplotype are functional: We previously analyzed *AS3MT* expression in whole blood (as a proxy of the arsenic-metabolizing organ, the liver) and found that expression was significantly altered in association with an increasing number of *AS3MT* protective alleles (Engström et al. 2011).

The present and historical arsenic concentrations in the drinking water of the Peruvian and HDGP populations included in this analysis are unknown. However, in Peru the arsenic levels in drinking water are generally much lower than levels in the northern part of Argentina, apart from some areas where mining activities have resulted in elevated levels during the last century (Bundschuh et al. 2008; Cooke and Abbott 2008). Still, the protective haplotype was more common in the Peruvian populations than in the HGDP populations, which may reflect movement of populations in the Andes Mountains throughout history and greater genetic similarity among more closely localized populations. Worth noting is that the Pima population from Mexico (included in HDGP) traditionally have lived in a region near the arsenic belt of Mexico, which has demonstrated increased levels of arsenic in drinking water probably for many generations (Camacho et al. 2011). The other Mexican population, the Maya, originates from a region far from the arsenic-rich one.

We do not have information about the correlation between the *AS3MT* haplotype and the arsenic metabolite phenotype for the Peruvian and the HDGP populations because urine samples were not available for these study groups. Elevated concentrations of arsenic in drinking water seem to be quite common in some areas of the Andes Mountains (Smith et al. 2006; Van Den Bergh et al. 2010) and several reports show that other Native American populations living in areas with historical arsenic exposure have efficient arsenic methylation. Hopenhayn-Rich et al. (1996) reported more efficient arsenic methylation in persons of Atacameño ethnicity (12.6% MMA in urine), compared with those of European ancestry on the Chilean side of the Andes highlands (17.2%, *p* < 0.001 between groups). Furthermore, Mexican populations of indigenous American ancestry that live in areas with historically high arsenic content have repeatedly been shown to have lower urinary percent MMA compared with populations of European ancestry (Gomez-Rubio et al. 2010, 2012; Meza et al. 2005). In addition, the frequency of protective *AS3MT* genotypes was higher in the Mexican populations than in the European populations. Furthermore, we have previously demonstrated comparable associations between the protective haplotype and arsenic metabolism in Argentina and in Bangladesh, although the haplotype is much less frequent in Bangladesh (Engström et al. 2011). Although the findings of this study suggest positive selection through the *AS3MT* gene for efficient arsenic metabolism, one needs to be cautious when interpreting the data. The fact that *AS3MT* is present in many organisms and conserved throughout evolution (Li et al. 2005) could also reflect unknown functions of AS3MT that are not related to arsenic metabolism. Further, genes that code other enzymes involved in human arsenic metabolism, such as the omega-class glutathione *S*-transferases, also may be affected by selection for efficient arsenic metabolism.

Because the protective haplotypes are found in diverse populations in East Asia and the Americas as well as in the European CEU HapMap group, albeit at lower frequencies than in the populations living in arsenic-rich areas in the Andes, selection for the protective variants is likely to have started from existing variation. This raises the possibility that selection may have driven the frequency of the protective haplotypes to high frequencies at several geographic locations.

There are a few well-known cases of selection in humans, for example, adaptation to lactase persistence has occurred independently in the same gene (lactase, *LCT*) in Africa and Europe (Tishkoff et al. 2007); variation in the copy number of the amylase gene (*AMY1*), improving the capacity to digest starch-rich diets (Perry et al. 2007); development of resistance to malaria (Kwiatkowski 2005); and the recently reported adaptation to living at high altitudes (Simonson et al. 2010). However, there is a lack of data on human adaptation to toxic compounds. Interestingly, selection may target a gene involved in arsenic metabolism and retention; many of the xenobiotic-metabolizing genes are highly polymorphic and demonstrate large variability in allele frequencies worldwide. This raises the possibility that human adaptation to environmental stressors is more common than previously thought.

## Conclusions

Populations living in environments with high arsenic exposure have significantly higher frequencies of genetic variants associated with efficient arsenic metabolism. Because the differences in frequencies were unlikely to be explained by population stratification, our data raises the possibility of acquired tolerance in humans to an environmental toxin. Further studies are needed to confirm these findings.

## Supplemental Material

(135 KB) PDF
