# Supplementary material for: Possible Positive Selection for an Arsenic-Protective Haplotype in Humans
Source: Environ Health Perspect. 2012 Oct 16;121(1):53–8. doi: 10.1289/ehp.1205504 (PMC3553437; doi:10.1289/ehp.1205504)
Supplement: (135 KB) PDF [file ehp.1205504.s001.pdf]

Supplemental Material

Possible Positive Selection for an Arsenic-Protective Haplotype in Humans

Carina M. Schlebusch, Cecil M. Lewis Jr., Marie Vahter, Karin Engström, Raúl Y. Tito, Alexandra J. Obregón-Tito, Doris Huerta, Susan I. Polo, Ángel C. Medina, Tom D. Brutsaert, Gabriela Concha, MattiasJakobsson & Karin Broberg

Table of contents

Supplemental Material, Table S1. 6-SNP *AS3MT* haplotypes

Supplemental Material, Table S2. 3-SNP *AS3MT* haplotypes

Supplemental Material, Table S3. *AS3MT* haplotype and median metabolite fractions among individuals in a) SAC and b) Close to Salta.

Supplemental Material, Table S1. 6-SNP *AS3MT* haplotypes

**Population**	**Region**	**ACTTAC**	**AGCTGT**	**AGCCGT**	**AGCTAC**	**AGCTAT**	**ACTTAT**	**ACTTGT**	**GGCTAT**	**ACCTAC**	**AGTTAC**	**ACTTGC**	**ACCTGT**
SAC	Native-American[a]	68.109	21.800	3.979	2.339	1.439	0.543	0.472	0.339	0.275	0.203	0.135	0.133
close to Salta	Native-American[a]	36.281	30.726	5.642	9.986	6.346	0.371	0.283	1.561	2.759	3.960	0.021	0.397
Peru	Native-American[a]	50.074	33.459	6.180	4.367	2.634	0.398	1.516	0.669	0.021	0.010	0.546	0.000
Maya	Native-American[b]	16.740	48.385	14.999	8.208	6.592	0.002	0.001	4.810	0.046	0.000	0.000	0.020
Colombian	Native-American[b]	13.166	62.549	0.000	8.256	8.875	0.002	0.001	7.123	0.000	0.000	0.000	0.000
Pima	Native-American[b]	12.204	49.645	24.997	6.485	6.600	0.002	0.002	0.000	0.001	0.001	0.055	0.001
CHB	East-Asia[b]	19.008	50.231	8.333	10.038	8.024	0.078	0.000	4.069	0.039	0.000	0.000	0.000
JPT	East-Asia[b]	22.189	49.357	6.221	12.181	6.804	0.030	0.001	3.033	0.000	0.000	0.000	0.000
Cambodian	East-Asia[b]	28.257	34.949	10.000	16.506	4.898	0.137	0.015	4.944	0.046	0.000	0.000	0.000
Daur	East-Asia[b]	16.272	51.772	14.954	8.633	8.227	0.000	0.001	0.000	0.001	0.000	0.000	0.000
Lahu	East-Asia[b]	31.392	42.470	0.000	18.373	7.474	0.000	0.001	0.000	0.057	0.110	0.001	0.000
Mongola	East-Asia[b]	25.100	29.071	22.221	13.530	4.261	0.001	0.000	5.443	0.002	0.099	0.000	0.000
Yakut	East-Asia[b]	8.143	63.574	6.667	5.157	9.756	0.002	0.001	6.440	0.000	0.000	0.000	0.000
Yi	East-Asia[b]	37.259	29.812	5.000	22.411	5.187	0.000	0.000	0.000	0.138	0.000	0.001	0.000
JPT + CHB	EastAsia[c]	23.333	45.556	8.889	12.778	7.222			2.222	0.000			
CEU	Europe[c]	5.833	56.667	4.167	15.833	0.000			11.667	5.833			

**Population**	**Region**	**ACCTAT**	**AGCCAC**	**ACCCGT**	**AGCTGC**	**ACTCAC**	**AGTTGT**	**GCTTAC**	**AGCCGC**	**AGTTGC**	**GGCTGT**	**AGCCAT**	**AGTCGT**
SAC	Native-American[a]	0.104	0.031	0.029	0.023	0.016	0.016	0.011	0.003	0.002	0.002	0.000	0.000
close to Salta	Native-American[a]	1.126	0.044	0.066	0.022	0.000	0.324	0.000	0.000	0.022	0.021	0.000	0.042
Peru	Native-American[a]	0.005	0.051	0.000	0.041	0.003	0.005	0.015	0.000	0.000	0.005	0.000	0.000
Maya	Native-American[b]	0.000	0.000	0.000	0.006	0.000	0.001	0.000	0.000	0.000	0.190	0.001	0.000
Colombian	Native-American[b]	0.000	0.000	0.000	0.007	0.000	0.001	0.000	0.000	0.000	0.019	0.000	0.000
Pima	Native-American[b]	0.000	0.000	0.000	0.005	0.000	0.001	0.000	0.000	0.000	0.000	0.002	0.000
CHB	East-Asia[b]	0.000	0.000	0.000	0.082	0.000	0.000	0.000	0.000	0.000	0.098	0.000	0.000

[Table S1, continued]

Population	Region	ACCTAT	AGCCAC	ACCCGT	AGCTGC	ACTCAC	AGTTGT	GCTTAC	AGCCGC	AGTTGC	GGCTGT	AGCCAT	AGTCGT
JPT	East-Asia[b]	0.000	0.000	0.000	0.005	0.000	0.059	0.000	0.000	0.000	0.092	0.029	0.000
Cambodian	East-Asia[b]	0.000	0.000	0.000	0.190	0.000	0.000	0.000	0.000	0.000	0.056	0.000	0.000
Daur	East-Asia[b]	0.000	0.000	0.000	0.094	0.000	0.001	0.000	0.000	0.000	0.000	0.001	0.000
Lahu	East-Asia[b]	0.000	0.000	0.000	0.068	0.000	0.055	0.000	0.000	0.000	0.000	0.000	0.000
Mongola	East-Asia[b]	0.000	0.000	0.000	0.158	0.000	0.000	0.000	0.000	0.000	0.112	0.001	0.000
Yakut	East-Asia[b]	0.000	0.000	0.000	0.033	0.000	0.001	0.000	0.000	0.000	0.227	0.000	0.000
Yi	East-Asia[b]	0.000	0.000	0.000	0.192	0.000	0.001	0.000	0.000	0.000	0.000	0.000	0.000
JPT + CHB	EastAsia[c]												
CEU	Europe[c]												

Abbreviations: CEU = CEPH (Utah residents with ancestry from northern and western Europe), CHB = Han Chinese in Beijing, JPT = Japanese in Tokyo, SAC = San Antonio de los Cobres.

[a] From this study, phased haplotype.

[b] Human genome Diversity Project (HGDP) populations from Jakobsson et al. (2008), phased + imputed haplotype.

[c] From hapmap 2 (www.hapmap.com), true haplotype.

Supplemental Material, Table S2. 3-SNP *AS3MT* haplotypes

**Population**	**Region**	**CTA**	**GCG**	**GCA**	**CTG**	**CCA**	**GTA**	**CCG**	**GTG**
SAC	Native-American[a]	68.678	25.807	4.148	0.607	0.378	0.203	0.163	0.017
close to Salta	Native-American[a]	36.652	36.411	17.937	0.304	3.885	3.960	0.463	0.388
Peru	Native-American[a]	50.490	39.686	7.722	2.062	0.026	0.010	0.000	0.005
Maya	Native-American[b]	16.742	63.580	19.610	0.001	0.046	0.000	0.020	0.001
Colombian	Native-American[b]	13.167	62.575	24.255	0.002	0.000	0.000	0.000	0.001
Pima	Native-American[b]	12.206	74.647	13.088	0.056	0.001	0.001	0.001	0.001
CHB	East-Asia[b]	19.085	58.743	22.131	0.001	0.039	0.000	0.000	0.000
JPT	East-Asia[b]	22.218	55.674	22.047	0.001	0.000	0.000	0.000	0.059
Cambodian	East-Asia[b]	28.394	45.194	26.348	0.015	0.046	0.000	0.000	0.000
Daur	East-Asia[b]	16.272	66.820	16.860	0.001	0.001	0.000	0.000	0.001
Lahu	East-Asia[b]	31.392	42.538	25.847	0.001	0.057	0.110	0.000	0.055
Mongola	East-Asia[b]	25.101	51.561	23.235	0.001	0.003	0.100	0.000	0.000
Yakut	East-Asia[b]	8.145	70.500	21.353	0.001	0.000	0.000	0.000	0.001
Yi	East-Asia[b]	37.259	35.003	27.598	0.001	0.138	0.000	0.000	0.001
JPT + CHB	EastAsia[c]	23.333	54.444	22.222		0.000			
CEU	Europe[c]	5.833	60.833	27.500		5.833			

Abbreviations: CEU = CEPH (Utah residents with ancestry from northern and western Europe), CHB = Han Chinese in Beijing, JPT = Japanese in Tokyo, SAC = San Antonio de los Cobres.

[a] From this study, phased haplotype.

[b] Human genome Diversity Project (HGDP) populations from Jakobsson et al. (2008), phased + imputed haplotype.

[c] From hapmap 2 (www.hapmap.com), true haplotype.

Supplemental Material, Table S3. *AS3MT* haplotype and median metabolite fractions [a] among individuals in a) SAC and b) Close to Salta.

	N	**% iAs**	**%MMA**	**%DMA**
**a) SAC**	256			
0 C-T-A copy [b]	24	15.2	9.9	75.4
1 C-T-A copy	106	13.7	8.8	77.2
2 C-T-A copies	126	11.7	7.0	81.0
p-value[c]		0.063	<0.001	<0.001
**b) Close to Salta**	10			
0 C-T-A copy	8	38.5	2.2	58.0
1 C-T-A copy	2	48.5	1.7	50.0
2 C-T-A copies	0	n/a[d]	n/a	n/a
p-value[c]		0.28	0.14	0.28

Abbreviations: DMA=dimethylarsenic acid, iAs= inorganic arsenic, MMA=methylarsonic acid, SAC=San Antonio de los Cobres.

[a] Men and pregnant women are excluded, since gender and pregnancy have been shown to influence arsenic metabolism.

[b] Number of *AS3MT* C-T-A haplotype copies (rs3740393, rs3740390 and rs10748835).

[f] P-values for haplotype (categorical variables for N of variant alleles) from regression analyses adjusted for urinary arsenic (natural log transformed). %iAs and %MMA were ln-transformed.

[d]N/a = not available (no individuals with that haplotype).

Reference

Jakobsson M, Scholz SW, Scheet P, Gibbs JR, VanLiere JM, Fung HC, et al. 2008. Genotype, haplotype and copy-number variation in worldwide human populations. Nature 451:998-1003.
